# Lack of detection of the Middle East respiratory syndrome coronavirus (MERS-CoV) nucleic acids in some *Hyalomma dromedarii* infesting some *Camelus dromedary* naturally infected with MERS-CoV

**DOI:** 10.1186/s13104-021-05496-w

**Published:** 2021-03-10

**Authors:** Maged Gomaa Hemida, Mohammed Alhammadi, Faisal Almathen, Abdelmohsen Alnaeem

**Affiliations:** 1 Department of Microbiology, College of Veterinary Medicine, King Faisal University, Al-Hufuf, Al-Ahasa, Saudi Arabia; 2 Department of Virology, Faculty of Veterinary Medicine, Kafrelsheikh University, Kafrelsheikh, Egypt; 3 Department of Public Health and Animal Husbandry, Veterinary Medicine, King Faisal University, Al-Ahasa, Saudi Arabia; 4 Department of Clinical Sciences, College of Veterinary Medicine, King Faisal University, Al-Ahasa, Saudi Arabia

**Keywords:** MERS-CoV, Dromedary camels, *Hyalomma dromedarii*, Nasal swabs, RNA, Real-time PCR, Transmission

## Abstract

**Objective:**

The Middle East respiratory syndrome coronavirus (MERS-CoV) is one of the zoonotic coronaviruses [Hemida Peer J 7:e7556, 2019; Hemida et al. One Health 8:100102, 2019]. The dromedary camels remained the only known animal reservoir for this virus. Several aspects of the transmission cycle of the virus between animals, including arthropod-borne infection, is still largely unknown. The main objective of the current work was to study the possibility of MERS-CoV transmission through some arthropod vectors, particularly the hard ticks. To achieve this objective, we identified a positive MERS-CoV dromedary camel herd using the commercial available real-time PCR kits. We collected some arthropods, particularly the ticks from these positive animals as well as from the animal habitats. We tested these arthropods for the presence of MERS-CoV viral RNAs.

**Results:**

Our results showing the absence of any detectable MERS-CoV-RNAs in these arthropods despite these animals were actively shedding the virus in their nasal secretions. Our results are confirming for the first the failure of detection of the MERS-CoV in ticks infesting dromedary camels. Failure of the detection of MERS-CoV in ticks infesting positive naturally infected MERS-CoV camels is strongly suggesting that ticks do not play roles in the transmission of the virus among the animals and close contact humans.

## Introduction

It has been almost eight years since the emergence of MERS-CoV in late 2012 in the Arabian Peninsula [3, 4]. Dromedary camels remain the sole animal reservoir of the virus until now [1, 2, 5]. Transmission of the MERS-CoV among animals within the same herd as well as between close contact dromedary camels populations was reported [6–9]. Detection of the MERS-CoV- and its nucleic acids (NA) in the nasal and rectal swabs were reported [7, 10–12]. New studies also reported the detection of the viral NA in the seminal plasma and breath of some positive MERS-CoV dromedary camels [13, 14]. Ticks are among the ectoparasites of many mammalian and avian species. There are two main types of tick, the hard ticks (Ixodidae) and the soft ticks (Argasidae). There are several known species of hard ticks infest dromedary camel. The Hyalomma species is the most prevalent type of hard ticks infesting dromedary camels in many countries across the world, including Saudi Arabia [15–17]. The parasite usually feeds on the blood of the affected animals or birds. There are four main stages in the tick's life cycle, starting with the eggs, the larva, and the adult. Ticks play important roles in the transmission of many pathogens, including bacteria, parasites, and viruses, between animals, including dromedary camels [16–21]. In 2007, Alkhurma Hemorrhagic Fever Virus (AHFV) was isolated from ticks (*Ornithodoros savignyi*) in western Saudi Arabia [20]. However, the roles of arthropods in general and ticks in specific in the transmission of MERS-CoV have not been studied yet.

## Main text

### Methodology

#### Dromedary camel herd description

**Fig. 1:**
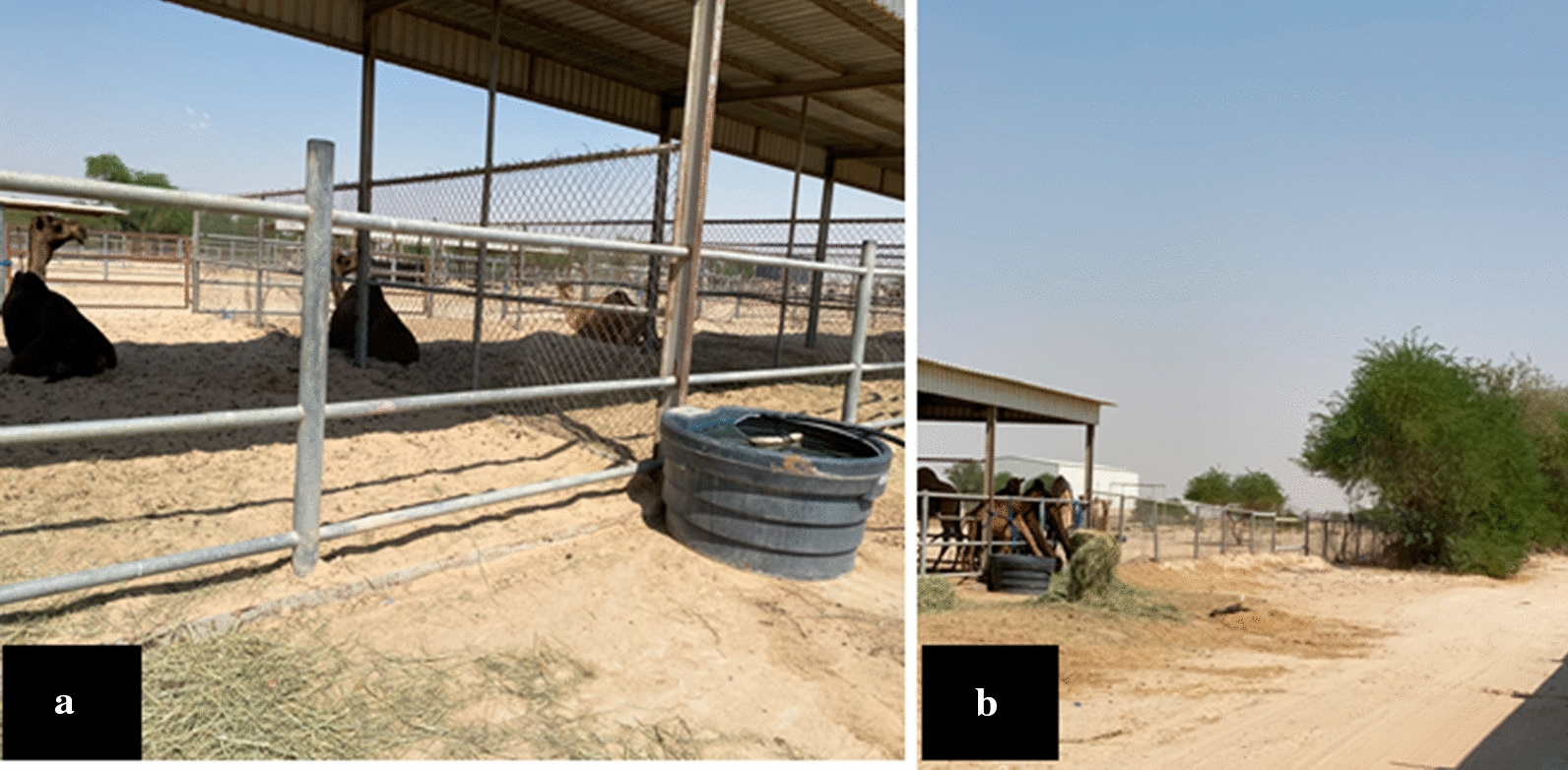
Dromedary camel herd habitat and organization **a** The male dromedary camels in separate wire fence compartments. **b** The female dromedary camels are mixed in a large wire fenced compartment

We conducted MERS-CoV molecular surveillance among one of the camel population in eastern Saudi Arabia belongs to the agriculture and veterinary research station, King Faisal University. The target animal population was 59 animals of both males and females raised in wire mesh fenced yards. The males live in separate pens (Fig. 1a) while the females live in a shared pen (Fig. 1b). Males only approach the females during the mating season. Males only approach females during the breeding (rutting season), which starts from late Oct to late April each year. Moreover, male camels displays an aggressive such as fighting between females for mating, and thus should be approached cautiously [22]. Animals usually shared common sources of food and water. We conducted a follow-up study on some of the animals in this herd that suffered from tick infestation.

#### Collection and processing of the nasal swabs

The nasal swabs were collected on a viral transport media, as previously described [10, 23], the cotton swabs were introduced deeply into the nasal openings of the target animals toward the back of the nasal passage. Each cotton piece of swabs should be soaked with a considerable amount of the nasal secretions of the animal. The collected swabs were transferred into the laboratory for further processing. Processing of these swabs was carried out by squeezing the cotton piece against the wall of the tubes then transferred the fluids into new sterile centrifuge tubes. The collected fluids were centrifuged at 5000 RPM for 5 min at (4 ºC). The supernatants stored at (− 80 ºC) until used.

#### Collection, identification, and processing of ticks from dromedary camels

Morphological identification of ticks was mainly conducted using the morphological keys of ticks, as previously described [24]. Ticks from each animal were collected in a separate sterile container. The collected ticks were placed on ice then transported to the laboratory. The ticks containing containers were placed at ( 80 ºC) for overnight. Morphological identification of the ticks was carried out by photographing the ticks using a dissecting light microscope as previously described [25]. Repeated cycles of freezing and thawing of ticks (at least three) were carried out by keeping the ticks at (− 80 ºC) for several hours, then thawed at room temperature. The lysis of the tick tissues was carried out as previously described [26].

#### The total viral RNAs extraction

The total viral RNAs were extracted from the nasal swabs and the tissue suspensions of ticks using the Qiagen viral RNA kits (RNeasy Mini Kit, Qiagen, Hilden, Germany). The process of these extractions was carried out as previously described [7, 14]. Simply, 140 μl per each sample was used to extract the total viral RNAs. The extracted RNAs were eluted in 50 μl of the elution buffer then were kept at (− 80 ºC) for further testing.

#### The real-time PCR, the RT-PCR, and sequencing

Testing of the nasal swabs, as well as the tissue suspensions from the collected ticks infesting this dromedary camel herd, was carried out as previously described [7, 10, 13, 14]. Simply, we used the commercially available real-time PCR MERS-CoV kits to test various samples for the presence of the viral nucleic acids. Samples were considered positive only when both targets (ORF1a and UpE) showing positive results. Meanwhile, we considered positive samples when (Ct ≤ 30). Confirmation of the identity of the positive MERS-CoV animals in the collected nasal swabs was carried out by amplification of the E gene as previously described [27]. The obtained sequences were deposited in the Genbank public domains.

### Results

#### Identification of some positive MERS-CoV infected dromedary camels and infested with Hyalomma dromedarii species of ticks

**Fig. 2:**
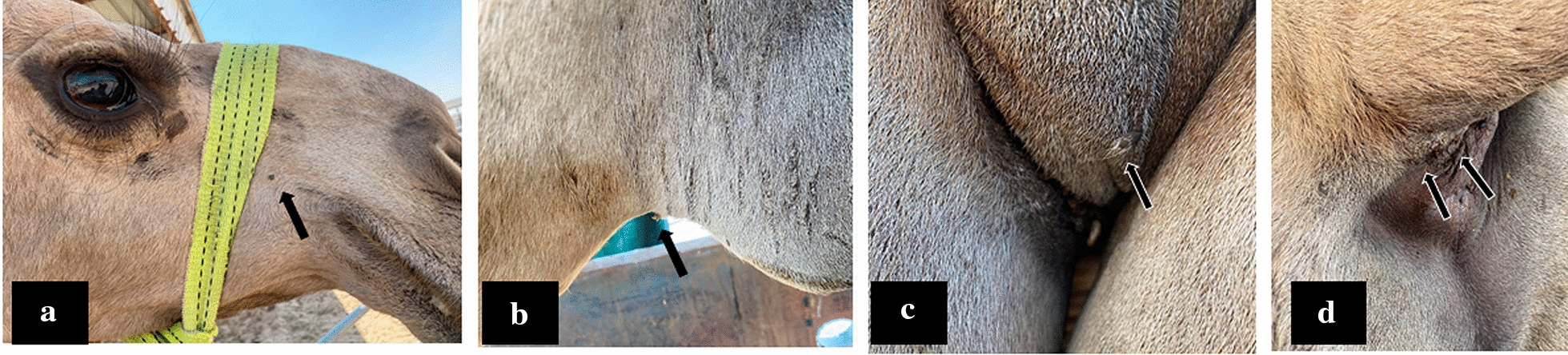
Distribution of ticks on the camel body **a** Ticks on the check of the dromedary camel. **b** Ticks on the prepuce of male dromedary camel. **c** Ticks on the testis of male dromedary came. **d** Ticks under the tail and around the anal opening of the dromedary camel

**Fig. 3:**
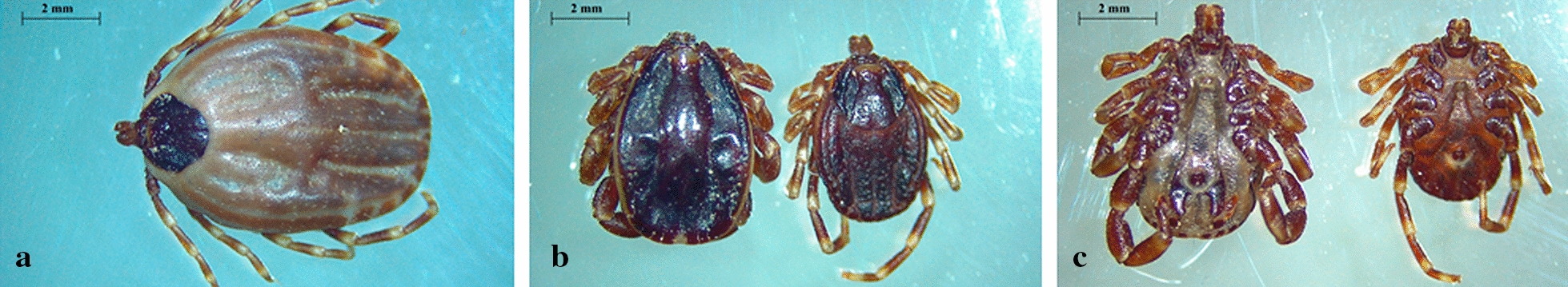
Morphology of the *Hyalomma dromedarii* infesting dromedary camels. **a** A dorsal view of female *Hyalomma dromedarii* tick engorged with blood **b** A dorsal view for a male (left) and female (right) *Hyalomma dromedarii.* **c** A ventral view for a male (left) and female (right) *Hyalomma dromedary*

We examined 45/59 of the animals in this target dromedary camel herd for the presence of tick infestation (Fig. 2). We found 36/45 examined animals infested with ticks by visual inspection (80%). The molecular surveillance showed 12/36 nasal swabs (33%) were MERS-CoV-NA positive. We selected 9/12 positive animals heavily infested with ticks and representing (15%) of the total herd for the collection of ticks. The selection of these target animals is based on the lowest Ct values (17–25) among the positive animals. To confirm the identity of the positive MERS-CoV animals, we sequenced some of the amplicons targeting the E gene. The obtained sequences were deposited in the Genbank (accession numbers: MW40645, and MW406455). Ticks in the target animals were distributed through different parts of the body of the infected animals especially on the checks, on the prepuce, testis of male animals, and under the tail and around the anal openings (Fig. 2a-d) respectively. The morphological identification of the collected ticks confirming that they belong to the *Hyalomma dromedarii* species of ticks (Fig. 3 a-c). A total of 43 ticks were tested for the presence of the MERS-CoV-RNAs in these nine animals. All the tested ticks sampled from the nine animals were negative for the MERS-CoV-RNA by the real-time PCR assay.

### Discussion

MERS-CoV is one of the most important zoonotic coronaviruses [1, 2]. Dromedary camels remain the only known reservoir of this virus until now [1, 6, 10, 13, 23]. Although the virus was identified more than eight years ago, many aspects of the transmission cycle and molecular pathogenesis require further studies. MERS-CoV is one of the respiratory viruses and proved to be transmitted from person to person on many occasions, including family clusters and some healthcare settings [28–31]. Meanwhile, evidence of camel-to-camel transmission within the same herd or closely related herds was also reported [7, 10, 32]. [9, 32–37]. Detection of MERS-CoV in the dromedary camel secretions and body fluids such as nasal secretions, rectal secretions, semen, and breath was also reported [10, 11, 13, 32]. Circulation of MERS-CoV in certain camel populations for a long time has been also reported [7, 9, 11]. High seroprevalence of MERS-CoV among the dromedary camel population in the Middle East and Africa was also documented [9, 32–37]. This high seroprevalence could be attributed to several factors, including the continuous circulation of the virus in some herds for a long time. This virus was shown to do repeated infection of the same herd over a period [7, 9]. Another possibility is the frequent introduction of newly infected animals to some herds [1, 5, 9]. This is in addition to the possibility of repeated infection through some arthropod born vectors such as mosquitoes and other ectoparasites such as ticks, flies, and Culicoides. However, the possibility of transmission of MERS-CoV through some arthropod vectors is not well studied yet. Tick infestation has been reported in dromedary camels for a long time [38–40]. Ticks infestation was also associated with the transmission of various pathogens, including bacteria, parasites, and pathogens to the dromedary camels [17, 19, 41, 42]. Both ticks and mosquitoes were implicated in the transmission of many important zoonotic viral diseases such as the Western Nile Virus (WNV) and the Crimean-Congo hemorrhagic fever virus [43]. A recent study was conducted in central Saudi Arabia to reveal the prevalence and molecular characterization of ticks, particularly the ixodid species in dromedary camels [15]. The same study confirmed that *Hyalomma dromedarii* was the most prevalent species of ticks in these animals [15]. We conducted molecular surveillance to monitor the presence of the MERS-CoV nucleic acids in the ticks infesting a positive MERS-CoV dromedary camel herd. The nasal swabs still the gold standard sample for the diagnosis of MERS-CoV in dromedary camels [5, 7, 34]. The selection criteria of the animals in the current study include, (1) animals should be infested with ticks at the time of sampling. (2) Meanwhile, ticks were collected from animals having different color coat based breeds available in this herd, including Magaheem, Sofr, Wodah, and Omani breeds. (3) We selected the animals with the lowest Ct values in their nasal swabs for MERS-CoV. This was to ensure these animals are active MERS-CoV shedders in their nasal secretions. The ultimate goal was to confirm that the tested animals were MERS-CoV infected and heavily tick-infested as well. Our results are clearly confirming the absence of any detectable MERS-CoV-NA in the ticks present on and in a camel herd in close proximity. These data are suggesting the lack of transmission of MERS-CoV through the *Hyalomma dromedarii* infecting these animals. The obtained results will enrich our knowledge about the transmission of MERS-CoV among dromedary camels. Further studies are needed for better understandings of various mechanisms of transmission of MERS-CoV among dromedary camels as well as from camels to humans.

### Conclusions

Absence of any detectable MERS-CoV nucleic acids in the tested arthropods, particularly the *Hyalomma dromedarii* ticks, strongly suggesting the lack of transmission of MERS-CoV through ticks.

## Limitations

The main limitations of the current study is the date representing only one actively shedding MERS-CoV dromedary camel herd infested with *Hyalomma dromedarii* ticks. A further large-scale study including several camel herds as well as a screening of various species of arthropods living in close contact with the dromedary camels such as Culicoides, mosquitoes, and Stomoxyes is needed for a better understanding of the roles of the arthropods in the transmission of MERS-CoV among animals.

## Data Availability

The generated data is available in the manuscript. The generated sequences are available to the public on the Genbank NCBI website as described above. The obtained sequences were deposited in the Genbank (accession numbers: MW40645, and MW406455).

## Abbreviations

MERS-CoV: Middle East respiratory syndrome coronavirus
NA: Nucleic acids
RT-PCR: Reverse transcriptase-polymerase chain reaction
RNA: Ribonucleic acid
AHFV: Alkhurma Hemorrhagic Fever Virus
WNV: West Nile virus
